# The Detection of Patients at Risk of Gastrointestinal Toxicity during Pelvic Radiotherapy by Electronic Nose and FAIMS: A Pilot Study

**DOI:** 10.3390/s121013002

**Published:** 2012-09-26

**Authors:** James A. Covington, Linda Wedlake, Jervoise Andreyev, Nathalie Ouaret, Matthew G. Thomas, Chuka U. Nwokolo, Karna D. Bardhan, Ramesh P. Arasaradnam

**Affiliations:** 1 School of Engineering, University of Warwick, Coventry CV4 7AL, UK; E-Mails: N.Ouaret@warwick.ac.uk (N.O.); M.G.Thomas@warwick.ac.uk (M.G.T.); 2 Department of Nutrition and Dietetics and the GI Unit, The Royal Marsden NHS Foundation Trust, Fulham Road, London SW3 6JJ, UK; E-Mails: box46@statacom.net (L.W.); J@andreyev.demon.co.uk (J.A.); R.Arasaradnam@warwick.ac.uk (R.P.A.); 3 MOAC Doctoral Training Centre, University of Warwick, Coventry CV4 7AL, UK; 4 University Hospital Coventry & Warwickshire, Coventry CV2 2DX, UK; E-Mail: chuka.nwokolo@uhcw.nhs.uk; 5 Department of Gastroenterology, Rotherham General Hospital, Rotherham S60 2UD, UK; E-Mail: bardhan.sec@rothgen.nhs.uk; 6 Clinical Sciences Research Institute, University of Warwick, Coventry CV2 2DX, UK

**Keywords:** electronic nose, FAIMS, fermentome, gastrointestinal toxicity, gut permeability, pelvic radiotherapy

## Abstract

It is well known that the electronic nose can be used to identify differences between human health and disease for a range of disorders. We present a pilot study to investigate if the electronic nose and a newer technology, FAIMS (Field Asymmetric Ion Mobility Spectrometry), can be used to identify and help inform the treatment pathway for patients receiving pelvic radiotherapy, which frequently causes gastrointestinal side-effects, severe in some. From a larger group, 23 radiotherapy patients were selected where half had the highest levels of toxicity and the others the lowest. Stool samples were obtained before and four weeks after radiotherapy and the volatiles and gases emitted analysed by both methods; these chemicals are products of fermentation caused by gut microflora. Principal component analysis of the electronic nose data and wavelet transform followed by Fisher discriminant analysis of FAIMS data indicated that it was possible to separate patients after treatment by their toxicity levels. More interestingly, differences were also identified in their pre-treatment samples. We believe these patterns arise from differences in gut microflora where some combinations of bacteria result to give this olfactory signature. In the future our approach may result in a technique that will help identify patients at “high risk” even before radiation treatment is started.

## 1. Introduction

The electronic nose (e-nose) was first conceived in the early 1980s [1] and has undergone continuous refinement ever since. Developed as a means of replicating the biological olfactory system, it does not identify specific chemicals within a complex mixture (as possible with, for example, gas chromatography and/or mass spectrometry—GCMS), but analyses the sample as a whole to create an “olfactory signature”. Such instruments have been applied widely in commerce and research, including for food and beverage quality, perfumes and process control [2,3]. Of increasing importance has been the application of this technology to the medical domain. The possibility of using the electronic nose for the identification and monitoring of disease has shown considerable promise. The detection of gas phase bio-markers from human biological output, be it breath, sweat, blood, urine or faecal matter, has been shown to identify a disease state. E-nose technology is close to real-time, can be operated without special gases, at room temperature and pressure, is non-invasive and could be produced at a financially acceptable cost for the medical profession. The range of diseases that e-nose technology has been applied to is considerable, ranging from lung cancer, brain cancer and melanoma to inflammatory bowel disease and even schizophrenia [4–9].

A more recent technological development is that of field-asymmetric ion mobility spectrometry (FAIMS) for monitoring complex odours. As with the electronic nose, FAIMS can be used for the real-time analysis of complex chemical components, looking at the total chemical composition of a sample. Such instruments use differences in the mobility of ionised molecules in high electric fields to provide a “mobility signature” of a complex sample. This mobility signature is in many ways comparable to the olfactory signature created by the array of chemical sensors in the e-nose. FAIMS instruments have found widespread use within the security sector [10], as they can detect large volatile molecules at extremely low concentration (e.g., explosives), but it has not as yet been used extensively within the medical field.

One medical area yet to receive attention by either e-nose or FAIMS technology is that of pelvic cancer patients, undergoing radiotherapy. The pelvis is a confined area bounded by bone and thick muscle, leaving only a fairly small central cavity. This is packed (from front to back) with the bladder, ovaries, uterus/cervix, the rectum, sigmoid colon (the lower part of the large bowel) and a variable number of small bowel loops. As a result, pelvic irradiation for a tumour affecting an organ inevitably injures the others. Almost all patients experience changes in bowel habits during their five to seven week course of radiotherapy. Up to 90% of patients report abnormal gastrointestinal symptoms of varying severity, termed “pelvic radiation disease” [11,12]. A wealth of data now supports the view that acute radiotherapy-induced damage is characterised by inflammatory processes. Maximum damage to the gastrointestinal mucosa occurs about two weeks into treatment [13]. However, whilst acute mucosal damage may then improve, the prevalence of moderate or severe chronic side effects can be as high as 50% [13,14]. Despite increasingly sophisticated radiotherapy planning and delivery, those patients who are at most risk of severe late problems cannot be predicted using normal medical measures. There is evidence that this high level of susceptibility may be due to differences in the composition and treatment-induced change of their gut bacterial populations [15,16]. Directly measuring the composition of these populations is incredibly difficult due to their location and huge variation in bacterial types, but we believe information in regard to its overall composition could be attained by looking at the gaseous emissions of bacteria within faecal samples. Thus, in an attempt to evaluate if it is possible to identify these “high-risk” patients, we have undertaken a pilot study to evaluate if either a traditional e-nose or a FAIMS instrument can identify differences in faecal gas emissions. Here we have taken samples before and after treatment from patients who have had a high toxicity and low toxicity response after treatment to investigate if there is a difference in these gas emissions. If feasible, it may be possible in the future to warn the clinician of patients that are likely to become seriously ill, thereby indicating who requires closer monitoring and/or possibly to be considered for an alternative treatment pathway. Such a patient-tailored approach offers a potential route towards minimising morbidity associated with this treatment.

## 2. Material and Methods

To undertake this study an in-house traditional electronic nose and a commercial FAIMS instrument has been used to analyse faecal samples from pelvic radiation patients.

### 2.1. Electronic Nose

Electronic noses are instruments that attempt to replicate the biological olfactory system, by investigating gas phase samples as a whole, instead of identifying specific chemicals within a complex gas mixture. In an electronic nose, the air above the sample (or headspace) is drawn into the electronic nose and is passed across an array of chemical sensors. The size of the array varies, but most are between six and 32 sensors. Each sensor of the array is broadly tuned to a chemical group, with overlapping sensitivity, but importantly, is different in some way from the rest of the array. As each sensor is dissimilar, the interaction between the sensor and the complex sample is unique within the array. Thus, an olfactory signature of this complex odour can be created. In most applications, some form of pattern recognition algorithm is applied to learn this olfactory signature and, when presented with the same profile, is able to recognize the odour.

The electronic nose used in this study is an in-house instrument developed at Warwick University. It contains an array of six metal oxide based sensors (high temperature resistive sensors), as well as six electrochemical sensors, a pellister (catalytic sensor), and an NDIR sensor (optical, to monitor CO_2_). A list of sensors and manufacturers is given in Table 1. These different sensor technologies were chosen for two reasons. First, it provides considerable diversity to the information that can be gathered from the array. Second, the sensors were selected to be broadly tuned to gases and vapours that we believe may be important products in the normal metabolic activity of the gut.

Due to the different types of sensors deployed within the instrument, it is useful to transduce each of the sensor outputs into a common form—in this case voltage. The six metal-oxide sensors were driven with a constant current source (100 μA) and the voltage across the sensor then amplified and monitored. The electrochemical sensors were attained with transmitter boards, containing a potentiostat and drive circuit to give a 4–20 mA output. This current was converted to a 0–10 V signal (note that the boards and sensors were calibrated for the gas concentration operating ranges defined by the manufacturer). The NDIR was also supplied with a transmitter board, giving a 4–20 mA output, which again was converted to 0–10 V. Finally, for the pellister a bridge circuit was manufactured with a differential output stage. Here the offset was trimmed to be zero in laboratory air and the differential output stage gain set to 20. The output voltage of all these sensors was measured using two National Instruments (USA) USB-6009 boards. The electronic nose was also fitted with a temperature and humidity sensor (Sensirion SHT-15, Staefa, Switzerland) to monitor the input sample. In addition to the sensors, the electronic nose was fitted with a pump with flow sensors, chamber temperature control, valves and dedicated software interface (written in LabVIEW 8.6, National Instruments, Woburn, MA, USA).

### 2.2. FAIMS Instrumentation

For this study a commercial FAIMS instrument was used, specifically a Lonestar (Owlstone, UK). FAIMS is a fairly recent technological development whereby separation occurs due to ionised molecules having different mobilities in high and low electric fields. Such instruments have a number of advantages, for example, they can use scrubbed air as the carrier gas, operate at room pressure (or above), and so no vacuum pump is required.

**Figure 1.**
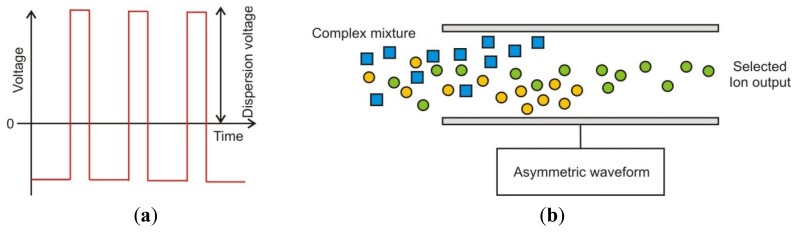
(**a**) Asymmetric waveform applied in FAIMS and (**b**) ions being separated by an asymmetric waveform.

In a FAIMS instrument the gas phase chemical input is first ionised (in our case using a Ni-63 source). These ionised molecules then enter an oscillating high electric field, whereby molecules zig-zag between two plates to which this electric field is applied. This waveform is “asymmetric”, thus a high positive voltage is applied for a short period of time and a low negative voltage is applied for a longer period. However, the integral of the voltage over a time period is zero. The waveform is stepped through a series of magnitudes and is called the “dispersion field” (DF). Below 200 V/cm the ions, on average, simply move back and forth between the plates but do not drift preferentially towards either one. At higher voltages in contrast, for example 5,000 V/cm or more, some ions within the complex mixture, on average move more in the direction of one plate. If an ion contacts the plate it loses its charge and is not detected as it exits the plates. Thus a balancing voltage (known as the “Compensation Voltage” or “CV”) is applied, which counteracts this drift. This compensation voltage can be set whereby only the drift from a specific ion is compensated for and will be detected. Thus by sweeping through a range of dispersion fields and compensation voltages a complex mixture of gases can be separated by their differences in mobility in high and low electric fields [17]. Figure 1 gives an example of the applied waveform for FAIMS and the separation of ions by the field plates.

### 2.3. Patients

Patients were recruited from a larger cohort undergoing long course (radical) radiotherapy with different end points and will be reported independently. Two groups were selected and stool samples from these patients were used for this study. The only criterion for inclusion was the degree of gastrointestinal disturbance between baseline and after four weeks of radiotherapy. This was measured by the IBDQ-B score (described below). The first group selected (group 1) were patients who had minimal toxicity as indicated by the least fall in the score; patients in the second group (group 2) were those who experienced the greatest fall in the score. Scientific and ethical approval was obtained from local Research Ethics and Scientific Committees of the Royal Marsden NHS Foundation Trust, London. Written informed consent was obtained from all patients who participated in the study. Previous studies examining the potential for identifying patients at risk of developing severe gastrointestinal toxicity during long course pelvic radiotherapy treatment have employed similar methodologies and patient numbers [15].

### 2.4. Samples

All data and samples were collected prospectively. Faecal samples (between 10 and 50 mL) were collected within 12 h of evacuation and immediately stored at −20 °C. Gastrointestinal toxicity was recorded using the validated IBDQ-B, which has been developed for use in monitoring disease activity in Ulcerative Colitis and Crohn's Disease [18,19]. The IBDQ-B is the bowel specific part of the questionnaire, which is a 10 question subset of the IBDQ. A maximum of seven points per question can be attained indicating no bowel toxicity (totaling a maximum score of 70) and a minimum score of 1 point per question (minimum score of 10 points showing most severe symptoms). A decline in score therefore represents increasing severity or frequency of symptoms. A decrease of more than six points has been shown to be clinically significant in a variety of settings [20,21]. Stool samples and IBDQ questionnaires were collected from all patients immediately before the start of radiotherapy (indicated as the baseline measurement) and after four weeks of treatment.

### 2.4. Analysis Methodology

Stool samples were thawed overnight to room temperature and then heated to 40 ± 0.1 °C in a dri-block™ heater for 1 h before the experiment. Ten mL of sample was aliquoted to a 25 mL sterilin bottle and 5 mL of deionized water added and manually shaken to homogenize the sample. Here, each sample was divided into two aliquots, one for e-nose, the other for FAIMS. Due to limited volume of sample, in some cases there was only enough sample for one aliquot. In these cases FAIMS analysis was prioritized over e-nose. The sterilin bottle lids were also modified to take pipe fittings for 3 mm PTFE tubing.

For e-nose experiments, laboratory air was used as the carrier gas with a measured humidity of 20 ± 2% r.h. (relative humidity). Before each experiment, laboratory air was pumped through the e-nose at a flow rate of 500 mL/min for 450 s to create a stable baseline. The stool sample line was then switched in and the headspace from the stool sample was passed into the instrument (also at 500 mL/min for 450 s), with a further 450 s of laboratory air to allow the sensors to recover back to their original baseline. Each sample was analysed in a random sequence and run twice. The sample humidity was measured for all experiments at 49 ± 5% r.h. with a random distribution between groups.

**Figure 2.**
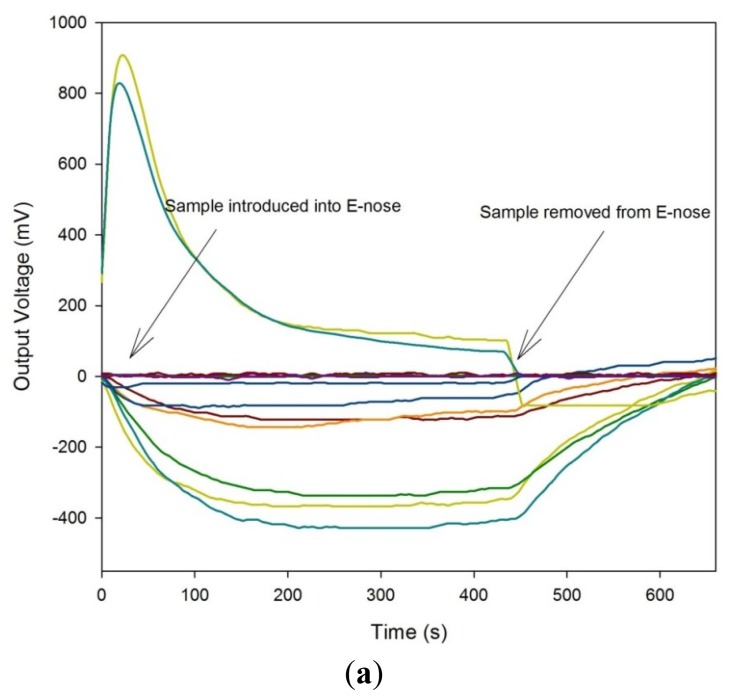

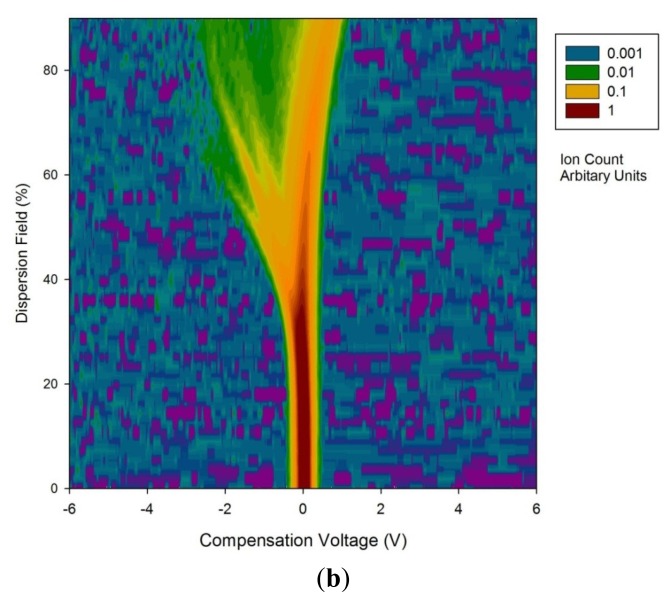
Typical output of the (**a**) Electronic Nose and (**b**) Owlstone FAIMS instrument.

For the FAIMS instrument, a similar approach was taken, though here, clean scrubbed compressed air was used as the carrier. The flow rate over the sample was 2 L/min, with the dispersion field scanned between 0 and 90% in 51 steps and the compensation voltage from −6 V to 6 V in 512 steps. Each analysis took typically 180 s and each sample was analysed twice. Figure 2 shows both a typical e-nose response (without legend for simplicity) and a FAIMS response to a stool sample.

E-nose feature extraction was performed using Multisens Analyzer (JLM Innovations, Tübingen, Germany). E-nose analysis used the change in voltage (delta V) as the input feature for analysis. Before exploratory analysis was performed, the data was pre-processed with a standardization routine, built into the Multisens software. Here the mean value of all the features was subtracted from all the features. Additionally, the features were then divided by the standard deviation of the features. This was done for two reasons, the first process allows only the differences between features to be evaluated, thus the absolute feature value (normally associated with intensity) was not considered. The second process ensures that each feature will receive equal weighting in the exploratory analysis. This method has been applied due to the very different sensor technologies used within the electronic nose. This data was then analysed by two different methods, Principal Component Analysis (PCA) and by Linear Discriminant Analysis (LDA). For PCA, the samples were analysed as a single group and then post-categorized, whereas for LDA the samples were pre-categorised according to clinical outcome (low and high toxicity). Principal component analysis is a method to convert multiple sensor responses from a group of observations (here patients) into a smaller number of artificial variables called principal components, where the total number of principal components is less that the number of sensors. This transformation produces results whereby the first principal component accounts for the largest variability in the data as possible. The succeeding principal components, in order, have the next highest variability and so on. These additional components are constrained in that they must be orthogonal to (*i.e.*, uncorrelated to) the proceeding components. Thus, the results produced by this transformation are unclassified, but also show if the different observations produce the most differences in the sensor responses. LDA is in some ways similar to PCA, but in this case, LDA attempts to find the optimum combination of sensors that best separates the differences between sample groups. So unlike PCA, which analyses samples based on the maximum difference, LDA looks for similarities within groups of samples.

**Figure 3.**
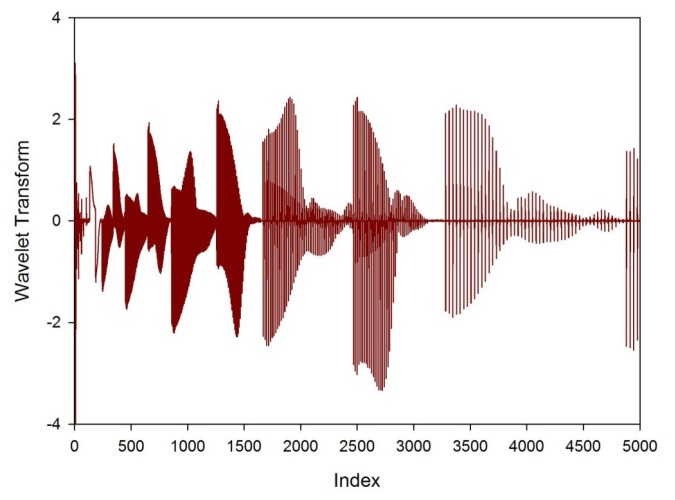
Wavelet transform of a low toxicity patient for elements 1 to 5,000.

FAIMS data was processed in Matlab (Mathworks Inc., Natick, MA, USA). As stated earlier, for each FAIMS measurement, the dispersion field was scanned between 0 to 90% and the compensation voltage between −6 V and 6 V. This produces 26,112 data points (as shown in Figure 1). In addition, both positive and negative ion counts were measured separately. Thus, the total number of data points was 52,254. To pre-process this data, a wavelet transform was applied (Daubechies D4). Wavelet transforms are a method used most commonly as a way of conditioning a signal into a more reduced form, before data compression. In simple terms, it projects the data on to a basis set of short waveforms (or wave-lets). Where the projections are large, this gives frequency and location information of this part of the original signal. Such techniques are incredibly good at separating signal information from noise. This is a natural method of pre-processing as the FAIMS output is a series of overlapping peaks, where one peak arises from molecules with a specific mobility. The wavelet transform gives information about this molecule, but also aids in discovering any additional molecules with similar, but not identical mobilities, by looking for differences in the frequency components on the signal. Figure 3, gives a section of the data after a wavelet transform had been applied. This was for a low toxicity patient, before treatment.

For analysis both the positive and negative ion count matrices from each sample were concatenated into a single 1D array 52,224 elements in length and then the wavelet transform applied. This produced a new 1D array, again 52,224 elements in size. Due to the large size of the dataset, it was required to identify elements that are suitable/useful in discriminating the different samples. To achieve this, thresholds were set for the within group (*i.e.*, low toxicity/high toxicity, pre and post treatment, thus four groups) scatter: (Σσ_i_)^2^ and the between group scatter: (σ_μ_)^2^/(Σσ_i_)^2^, to identify dimensions for selection (σ_i_: the standard deviation of the dimension in question within the group i, and σ_μ_ was the standard deviation of the means of the dimension under test between classes). The exponents change the form to reflect that employed in Fisher Discriminant Analysis (FDA). FDA is almost identical to LDA, except that it does not make some of the assumptions used in LDA (such as normal distributed classes). It was discovered that this gave better discrimination for the methods and data captured by FAIMS.

This approach gave a two dimensional input parameter space to control the separation algorithm. This space was explored as follows. For each point in the input space 20 test sets (each containing one high toxicity response and one low) was re-classified. These test sets were not used in the identification of dimensions or the implementation of the FDA. This exploration identified regions in the parameter space where re-classification exceeded the 65.8% that would be expected for random re-classification (two standard deviations from the mean), and for which the success of re-classification was robust to perturbation in the parameters while changing the number of dimensions identified.

## 3. Results and Discussion

**Figure 4.**
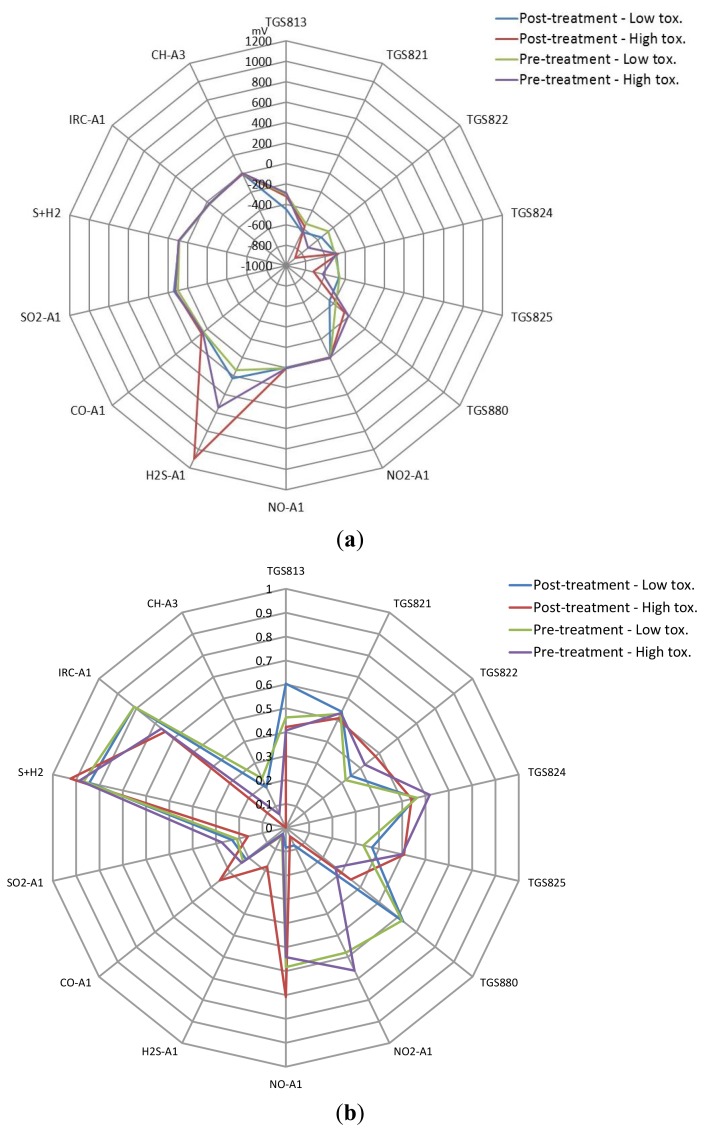
(**a**) Averaged e-nose sensor responses for different sample groups (axis is change in output voltage) and (**b**) Normalized responses.

In total, 23 patients were investigated, 11 in the low toxicity group and 12 in the high toxicity group. The sample number, age, male/female ratio, radiotherapy dose and symptom burden (IBDQ-B score) analyzed by e-nose and FAIMS is shown in Table 2. The patients were of a similar age in both groups and had a similar dose of radiotherapy, including fractionation and duration of treatment (data not shown). By selection, the fall in the IBDQ-B score was only slight in the low toxicity group, but was marked and significant in the high toxicity. Numbers of samples per instrument differs slightly due to available volume of biological matter. Figure 4 shows the average e-nose sensor responses to the four groups from Table 2 (Figure 4(a)—raw values and Figure 4(b)—normalized before averaged). Here, only one set of data was used for these figures, even though the samples were run twice. This was done as we have previously noticed sample depletion with running samples using a dynamic sampling approach, thus the sensors are exposed to different gas profiles between tests.

**Figure 5.**
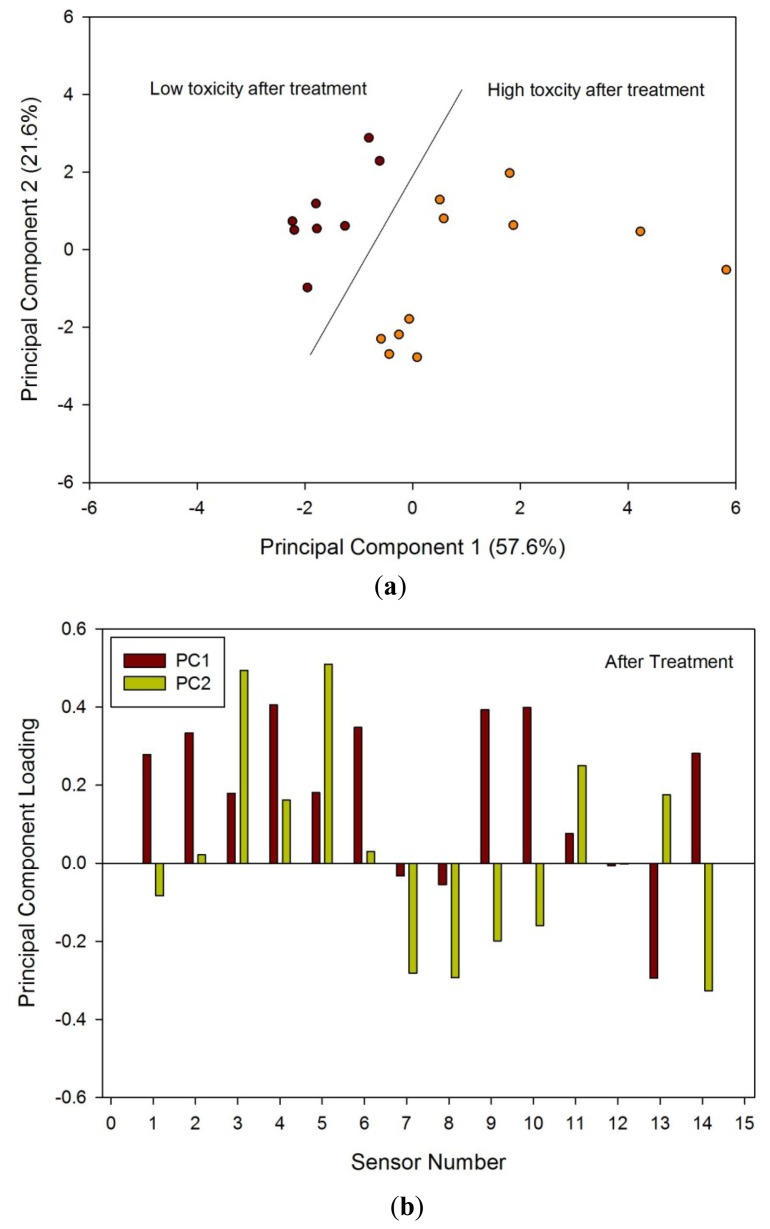
(**a**) PCA results from e-nose data and (**b**) loadings for samples taken four weeks after treatment referenced to Table 1.

Samples after radiotherapy were analysed first, as the differences between these groups were likely to be greatest. Figure 5(a) shows the PCA of the two groups after treatment; there was a clear separation between the low and high toxicity groups. Figure 5(b) shows the loadings of this analysis, indicating that a large number of sensors contributed to the variability. LDA was also performed that again showed clear separation between the two groups (hence the results not shown here).

**Figure 6.**
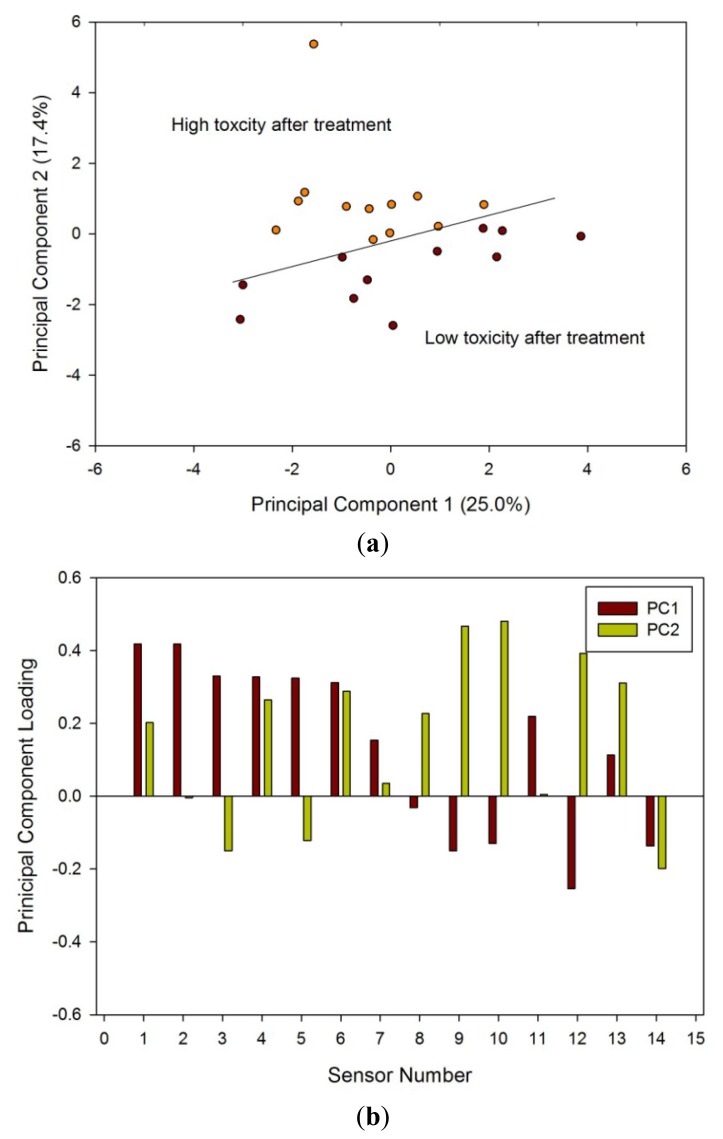
(**a**) PCA results from e-nose data and (**b**) loadings for samples taken before treatment; categorized on their post-treatment toxicity referenced to Table 1.

Analysis of the pre-treatment samples by PCA Figure 6(a) and by loadings Figure 6(b) indicated a difference between those who ultimately went on to have high or low toxicity. The differences, though statistically significant, were less striking.

**Figure 7.**
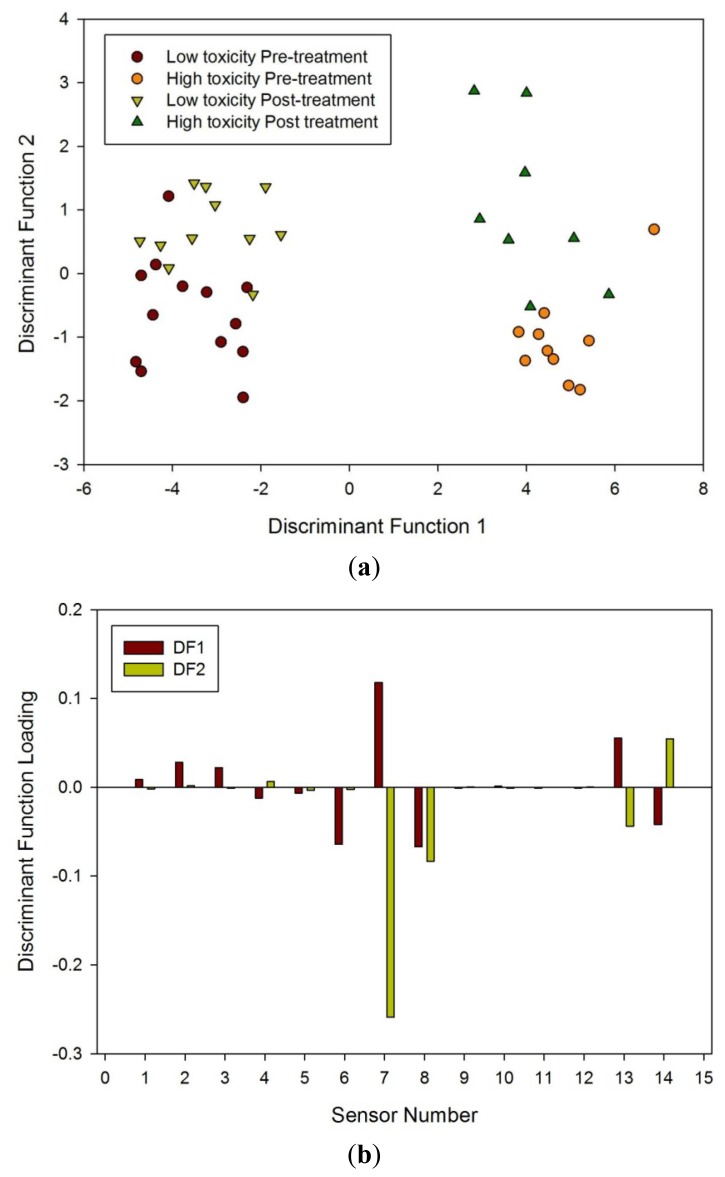
(**a**) LDA of all four groups using the e-nose and (**b**) loading plot associated with LDA referenced to Table 1.

Finally, LDA was undertaken for all four groups. Though LDA could clearly separate high and low toxicity, pre and post treatment, it could not separate all four groups when analyzed together, as shown in Figure 7. What it does suggest is that there is a significant difference between those susceptible to radiation damage to those who are less so.

Although the sample numbers were small, a KNN (K-nearest neighbour) algorithm was applied to the electronic nose data to evaluate if it was possible to classify each sample correctly. Using n − 1 as the training set (where n is the number of samples), it was possible to correctly classify 22 out of 23 samples correctly (pre-treatment). When 10 samples were moved at random from the reference set to the test set, it was still able to correctly classify 80% of the samples consistently.

**Figure 8.**
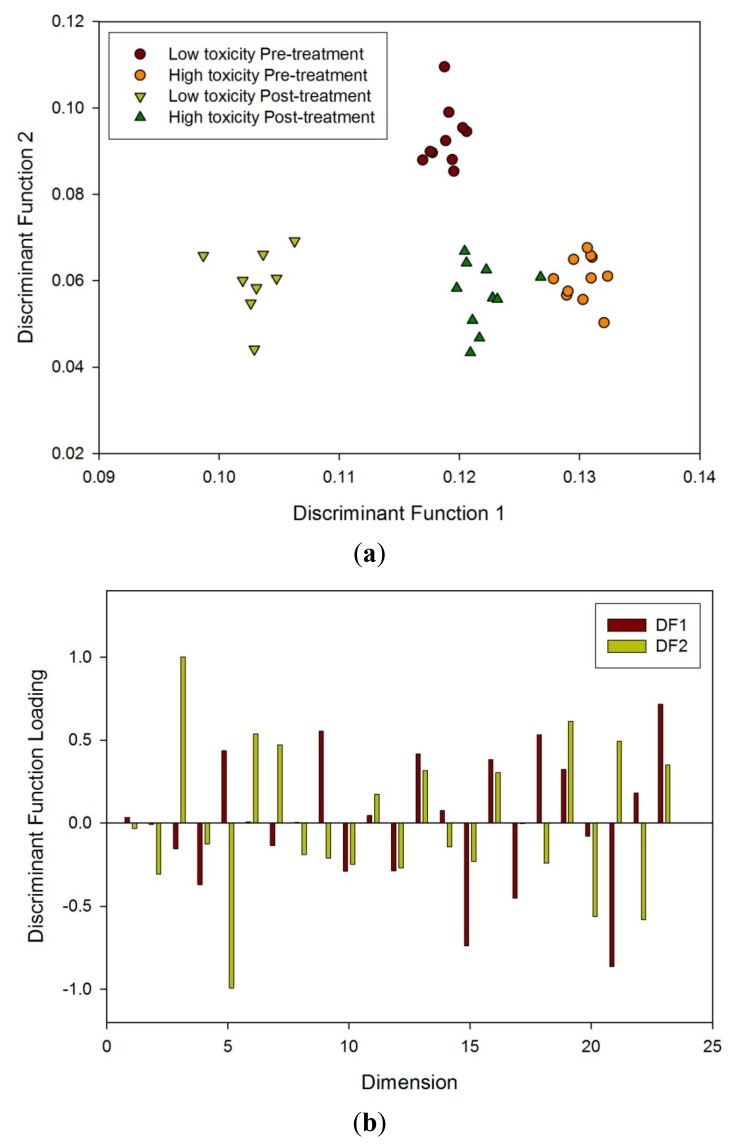
(**a**) FDA of FAIMS data to all four groups and (**b**) loading plot associated with FDA using dimensions created in the analysis described earlier.

FAIMS analysis was done first from post radiotherapy samples as greater differences were expected, then on the pre-treatment samples and finally as all samples together. It was discovered that FDA could separate the different groups in all three cases. Figure 8(a) shows the FDA result from the FAIMS data from all the samples. Thresholds of 0.16 on the between class scatter and 0.12 on the within class scatter were imposed for the selection of dimensions. This identified 23 dimensions which were used for the FDA. Taking one sample out (n − 1) and re-performing the FDA, the accuracy of reclassification was in excess of 90% for both groups. Figure 8(b) shows the components of the projection vector identified and indicate that a large number contribute significantly to the classification.

### 3.1. Discussion

Pelvic radiotherapy is a highly invasive procedure that frequently causes gastrointestinal side-effects as a result of tissue damage. Currently there is no reliable means of identifying those patients who will have a severe reaction. Our results indicate that the difference in odour profiles distinguishes patients severely affected from those with milder symptoms, using the patients themselves as the control group for this pilot study. Furthermore, this difference may be measured before treatment. As shown in the loading plot Figures 5(b) and 6(b) volatiles and gases obtained from stools collected before and after radiotherapy activate different sets of sensors, a clear indication of a change in the gas chemical profile. The gaseous outputs reflect the pattern of chemicals released by the fermentation of undigested fibre by resident gut microbes, principally within the colon. The change observed, we hypothesise, results from radiation which perturbs the balance between the many species that together make up the gut microflora. Of note is that the LDA plot of the toxicity groups Figure 7(a) is unable to separate the before and after treatment for either toxicity group, but clearly distinguishes those patients who will be severely affected from those less so. More interesting, the loading plot Figure 7(b) shows that these groups are separated by gases/vapours that could be associated with the fermentation process, specifically hydrogen, methane/combustible, carbon dioxide, nitrogen dioxide and volatile organic compounds.

Our pilot data do not allow us to comment upon how radiation affects microbial behaviour, either by direct action or indirectly through resultant mucosal inflammation. We are also unable to comment if the altered microbial balance is a passive reflection of mucosal inflammation or whether it actively worsens it. Nevertheless our results suggest we now have the potential to identify patients “at risk” of severe radiation-induced gastrointestinal damage before starting such treatment.

Our speculation on the role of microbes is plausible and has a precedent in Crohn's disease, a gut disease characterized by inflammation—some patients affected mildly but others severely. Using electronic noses we have identified that the patterns of volatiles and gases emitted from the urine of such patients has a different pattern to that in health, signifying a difference in the respective resident microbial flora; furthermore, the pattern changes again when Crohn's activity flares up [22]. An interesting and very relevant recent finding is that the spectrum of microbial profile is reduced in Crohn's disease. Hence there is growing interest in the possibility that altered gut microbes may be more intimately linked with the disease than was at first thought.

Studying the microbial flora is difficult as many species cannot (as yet) be cultured; hence complex molecular methods are required, which currently remains at the research level. Studying instead the volatiles from gut fermentation provides a more accessible insight into microbial behaviour. Gas chromatography (GC)-mass spectrometry (MS) can identify individual volatiles but is complex and expensive; examining the overall pattern of the “fermentation signature” instead, using electronic noses or FAIMS, is more accessible.

Using the electronic nose to recognize odour patterns we were able to separate the “high risk” group from those at lower risk. We achieved the same separation with FAIMS, proof of principle that the investigation of movement patterns of the volatiles can be used in such medical investigations.

FAIMS is an interesting technology for future development. It has the advantages of high sensitivity (compared with traditional electronic nose instruments), can operate at room temperature and does not require special gases (as with GC or GC-MS). Its high sensitivity is however also a limitation as contamination reduces performance. Its other limitation is the very large datasets it generates, which in our study was 50,000 data points per sample. However, our technique of wavelet transformation combined with FDA allows us to handle such large data. FAIMS is still in its infancy but the technique is powerful; it therefore seems likely that as research continues the instruments will improve and evaluation of large datasets will allow full comparison with traditional electronic nose instrumentation.

## 4. Conclusions

Radiotherapy as a treatment for cancer has attained a high level of success, but can have major side-effects. For those patients receiving pelvic radiotherapy there is a marked variability in the level of gastro-intestinal toxicity they may suffer. To inform treatment pathway, it would be beneficial to identify those patients who will have the most significant reaction and then alter their treatment accordingly—a patient tailored approach. In this pilot study, we have taken stool samples from patients before and four weeks after receiving radiotherapy. Of these patients a subset of 23 patients, who had the most significant reaction and those with the minimum reaction, were analysed by an in-house electronic nose and a FAIMS instrument. In this instance patients acted as their own control, as the aim of our study was to determine and track any changes (gases/volatiles) which could be detected. Using PCA for e-nose data and a wavelet transform followed by FDA for FAIMS data, the results indicated that it was possible to detect differences in odour profile of stool samples after treatment. More interestingly, these patients can be separated using these analysis techniques before radiotherapy. This suggests that there are substantial differences in stool composition between these two groups, prior to radiotherapy, indicating a difference in indigenous bacterial micro-flora between patients. Future work will look at increasing the size of the sample groups, analysing patients between the two extremes, undertaking GCMS (which is particularly challenging for fecal samples due to the large number of different chemicals involved) and instigating a study into the bacterial differences.

## Figures and Tables

**Table 1.** Sensors deployed within the electronic nose, with descriptions. Number is brackets is sensor number within the instrument.

**Metal Oxide Sensors** **(Figaro Jpn)**	**Electrochemical Sensors** **(Alphasense/Sensors Direct, UK)**	**Others** **(Alphasense, UK)**
TGS813—Low pressure gas (1)	NO_2_-A1—Nitrogen dioxide (8)	IRC-A1—Carbon dioxide (7)
TGS821—Hydrogen (2)	NO-A1—Nitrous oxide (9)	CH-A3—Flammable gases (13)
TGS822—Organic solvents (3)	H_2_S-A1—Hydrogen sulphide (10)	
TGS824—Ammonia (4)	CO-A1—Carbon monoxide (11)	
TGS825—Hydrogen sulphide (5)	SO_2_-A1—Sulphur dioxide (12)	
TGS880—Volatile vapours (6)	S+H_2_—Hydrogen (14)	

**Table 2.** Baseline demographic characteristics and treatment features: Groups 1 (low toxicity) and 2 (High toxicity).

**Feature**	**Electronic Nose Analysis**		**FAIMS Analysis**	
Least Toxicity:	Pre-treatment	Post-treatment	Pre-treatment	Post-treatment
Number of patients (Male:Female)	N = 11 (9:2)	N = 8 (7:1)	N = 11 (9:2)	N = 10 (8:2)
Mean Age (s.d.):	71.9 (4.8)	70.9 (5.1)	71.9 (4.8)	71.3 (4.5)
Mean Radiotherapy dose	61.8	63.3	61.8	61.0
Mean IBDQ Scores (s.d.)	67.4 (2.6)	67.5 (2.7)	67.4 (2.6)	67.3 (2.7)
High Toxicity:	Pre-treatment	Post-treatment	Pre-treatment	Post-treatment
Number of patients	N = 12	N = 11	N = 12	N = 10
(Male:Female)	(9:3)	(8:3)	(9:3)	(8:2)
Mean Age (s.d.):	69.8 (11.3)	69.1 (11.1)	69.8 (11.3)	71.8 (7.3)
Mean Radiotherapy dose	60.4	59.5	60.4	61
Mean IBDQ Scores (s.d.)	68.8 (1.7)	48.5 (7.4)	68.8 (1.7)	49.8 (6.2)
